# Effect of public-private interface agency in Patna and Mumbai, India: Does it alter durations and delays in care seeking for drug-sensitive pulmonary tuberculosis?

**DOI:** 10.12688/gatesopenres.13113.1

**Published:** 2020-04-09

**Authors:** Sanchi Shah, Shimoni Shah, Sheela Rangan, Sonukumar Rai, Eunice Lobo, Swaran Kamble, Yatin Dholakia, Nerges Mistry

**Affiliations:** 1 The Foundation for Medical Research, Mumbai, India

**Keywords:** Public private interface agency, DSTB, spoke and hub, pathways, duration and delays

## Abstract

**Background: **Public–private interface agency (PPIA) intervention models in Patna (E. India) and Mumbai (W. India) for pulmonary drug-sensitive (DS) tuberculosis (TB) patients were evaluated over 2 years after maturity to examine effect on reduction of patient pathways and retention.  The models engaged private providers, diagnostic facilities and pharmacies into an effective network providing free diagnostic tests and treatment.

**Methods: **A population-based retrospective study was undertaken to assess effectiveness of the PPIA model in care pathways of 64 (Patna) and 86 (Mumbai) patients through in-depth interviews conducted within 6 months of initiation treatments to identify types and facilities accessed, duration to diagnosis and treatment. Median durations based on facilities accessed were statistically analysed.  Comparisons were made with baseline values and endline pathways of patients accessing PPIA engaged/non-engaged facilities in private and public sectors.

**Results: **Compared to non-engaged facilities, persons accessing engaged facilities at first point-of-care had shorter pathways (Mumbai: 32 vs 43 days), (Patna: 15 vs 40 days).  Duration for first care-seeking was considerably shorter for patients accessing PPIA in Patna and for both engaged and non-engaged private facilities in Mumbai (4 days).  Whilst PPIA engaged facilities diagnosed more cases than others, the RNTCP in Mumbai provided diagnosis early.  There was good retention of patients by PPIA-engaged (1 ^st^) facilities (90% post-diagnosis in Patna) but this was affected by the hub-spoke referral system in Mumbai (13%). Second diagnosis is a common feature in Mumbai.  The spoke-hub model in Mumbai contributed considerably to treatment delay; PPIA-engaged providers were better at retaining patients post treatment initiation 11/25 (44%).

**Conclusion: **PPIA-engaged facilities, accessed at onset, result in marked reduction in pathway durations.  Such initiatives should engage a critical mass of competent providers, proximal investigation facilities with enhanced disease awareness and literacy efforts amongst communities.  Patient movement should be minimized for early treatment and retention.

## List of abbreviations

RNTCP: Revised National TB Control Programme; PPIA: Public Private Interface Agency; WHP: World Health Partners; CXR: Chest x-ray; CBNAAT: cartridge-based nucleic acid amplification test; PATH: Partnership for Appropriate Technologies in Health; HH: household; DS: drug sensitive; PPIA-E facility: PPIA-engaged facility; PPIA–NE facility: PPIA-non engaged facilities; DR: drug resistant.

## Introduction

The National Strategic Plan of India envisages ending tuberculosis (TB) by 2025, ten years ahead of the END TB Strategy proposed by the WHO ^1^. It is estimated that 2.8 million new cases occur annually, of whom nearly a million are not being identified and notified ^2^. The Revised National TB Control Programme (RNTCP) employs various strategies to identify these cases early and treat them appropriately, in order to reduce disease transmission and meet these ambitious targets ^3^.

Identifying symptomatic individuals in the community and bringing them on appropriate treatment is resource intensive and has had limited success ^4, 5^. The RNTCP has attempted involvement of the private sector by employing various strategies ^6^ over two decades with varying success ^7^. The 14-city Public–Private Mix (PPM) projects initiated in 2003 had shown an overall increase in the number of cases notified. The Mahavir project in Hyderabad and the Kannur initiative in Kerala showed a 20 to 100% improvement in case detection ^6^. While cost-effectiveness studies conducted on some of these PPM initiatives have found them to be expensive, it was concluded that these costs are likely to reduce the societal cost of shopping for private health care and improve standards of care ^8^.

Several studies undertaken in recent years show delays in first care seeking, diagnosis and TB treatment among patients accessing both public and private sectors ^9– 12^. In order to improve access to quality of TB care and thereby reduce delays in patient care pathways, a public–private interface agency (PPIA) intervention was implemented in Mumbai and Patna. It was hypothesized that if a patient reached a PPIA engaged formal facility at the first point of care, there would be less shopping between facilities, faster and more accurate TB diagnosis and quicker treatment initiation.

World Health Partners (WHP) implemented a PPIA model in Patna district from May 2014 to June 2016 that engaged and sensitized private providers, brought diagnostic facilities, and pharmacies into a referral network to provide adequate care, including free diagnostic tests like chest X-ray (CXR), smear microscopy, and cartridge-based nucleic acid amplification test (CBNAAT), facilitated TB notification and free TB treatment for notified patients and ensured treatment adherence support. The model engaged 71% (643) of the targeted 912 licensed formal providers and 71% (688) of 972 pharmacies (WHP Personal Communication).

In Mumbai, Partnership for Appropriate Technologies in Health (PATH) implemented a hub and spoke model from September 2014 to June 2016, which engaged private providers, both allopaths and non-allopaths to provide free diagnostic tests (X-ray and CB-NAAT), first line anti-TB treatment drugs and monitoring of treatment outcome. The spokes consisted of stand-alone clinics or OPD facilities whose main aim was to identify presumptive TB patients and refer them to hubs. Hubs were facilities which had a physician/chest specialist along with X-ray facility and a dispensing unit. They were assigned to provide diagnostic tests and prescribe treatment. A total of 7,396 doctors, 2,773 chemists and 747 laboratories were mapped in 15 high burden TB wards of the city, of which 51% doctors, 34% laboratories, and 13% chemists were prioritized and engaged in the PPIA intervention (PATH Personal Communication).

At 2 years after the baseline study in Patna ^9^ and 2 years and 8 months after in Mumbai ^10^, end-line studies were conducted in the same areas to study the impact of the PPIA on patient TB care pathways. Although many earlier studies have looked at delays in diagnosis and treatment among TB patients, these studies, for the first time assessed a specific intervention in terms of its effect in reducing delays in TB diagnosis and treatment.

## Methods

### Study design

The end-line studies were conducted between August 2016 and January 2017 in Patna and between March 2017 and July 2017 in Mumbai after the PPIA intervention had been in place for 27 months in Patna and 29 months in Mumbai. These studies were conducted to assess the impact of the intervention in both cities. The end-line studies were population-based, two-stage, retrospective studies conducted in the same study areas as the baseline studies in both cities ^9, 10^. The first stage surveys conducted by a contracted survey organization in both cities involved a primary cross-sectional household (HH) survey to identify patients who had been diagnosed and initiated on TB treatment (available as *Extended data* ^13^). Within a week of identification, these patients were contacted by a trained team of researchers from FMR, and those who met the inclusion criteria and were willing to participate were included in the second stage of the study. The inclusion criteria were as follows: a) patients with only pulmonary TB, b) patients diagnosed as drug-sensitive (DS) and initiated on first-line TB treatment in their respective cities, c) patients who were initiated on treatment not earlier than six months from the time of interview. The number of participants was selected through convenience-based sampling.

The second stage consisted of in-depth interviews of willing patients, using a semi-structured interview schedule to document their TB care pathways (interview guide available as *Extended data* ^13^). Written informed consent was obtained from all patients willing to participate in the study. Consent was sought for participation in the interview, digital audio recording and note keeping of the patients’ responses, reviewing of patient’s TB care seeking-related documents and permission to publish data in any report, journal, etc. In case of minors, consent was obtained from their parents/guardians/carers.

Interviews were conducted at a location and time preferred by each participant by trained health researchers from FMR largely in patient’s homes. Family members were sometime present at the time of the interviews, which extended from 45 minutes to 1 hour. No repeat interviews were carried out. Patients were confirmed as DS based on their test results and treatment regimen at the time of interview. The end-line interview assessed the time taken by patients from onset of TB symptoms until the current/most recent treatment initiation (unlike the baseline study where only first treatment initiation was considered) ^9, 10^, along with the type of facilities accessed.

Information provided by patients during the interview was verified as far as possible with reports prescription or other documentation provided by the patient.

### Ethics approval and consent to participate

The study received ethical declaration from the Institutional Ethics Committee (IEC) of the Foundation for Medical Research (vide IEC no. FMR/IEC/TB/01/2013). Written informed consent was obtained from all patients willing to participate in the study. In case of minors, consent was obtained from their parents/guardians/carers.

### Data management

Quantitative data were filled on structured physical formats by the researchers at the end of the interview. For the purpose of quality check, three levels of verification were conducted by listening to recorded interviews. First, each researcher team cross-checked the quantitative data forms of interviews conducted by another research team. Subsequently 25% of interviews were cross-checked by senior researchers for errors, and finally a random set of 10% of interviews were checked by the consultants on the study. On completion of quality checks, the data were entered in CSPro v5. Open ended questions were coded and maintained in a codebook in an excel format. These data were then imported into SPSS v.19 (SPSS, Inc., Chicago, IL, USA) for analysis.

### Operational definitions

***Facility classification***

**1. Public Facility:** Facilities for control of TB which are run by the Municipal Corporation of the respective city or the state government under the RNTCP.

**2. Private Facility:** Facilities which are run by private allopaths, non-allopaths, informal health providers and chemists.

**3. PPIA-engaged Facility (PPIA-E Facility):** Facilities which consented to engage with the PPIA. In Mumbai, only private providers were a part of the PPIA intervention whereas in Patna, PPIA-E facilities included both public and private facilities.

**4. PPIA-non engaged Facility (PPIA–NE Facility):** Private facilities which were not engaged with the PPIA initiative.

The total pathway was divided into three components for computing durations:

- First care seeking duration: Time interval between onset of symptom and first seeking care(duration of 15 days or more was considered as a first care seeking delay) ^14^.
- Duration to TB Diagnosis: Time interval between first seeking care and first TB diagnosis by a facility (duration of 15 days or more was considered as a diagnostic delay) ^15^.
- Duration to TB Treatment initiation: Time interval between first TB diagnosis and initiation of TB treatment for the first time. In case of multiple diagnostic episodes prior to treatment initiation, the first diagnostic episode was considered for computation. (duration of more than 7 days was considered as a treatment initiation delay) ^15^.

### Data analysis

Facilities were determined as PPIA-E based on the names and addresses provided by patient interviews and documents, which were compared to the list of engaged facilities provided by PPIA in Patna and Mumbai respectively.

The total time taken from onset of TB symptoms to first care-seeking and until initiation of first line treatment was estimated by dates collected for various events and presented as medians and means (in days).

The themes for analysis were identified in advance. The median pathway durations of patients accessing different types of facilities at baseline and end-line were compared until the point of first treatment initiation to see if any difference was seen in pathways to TB care following the PPIA intervention in both cities. The prime comparison comprised of assessing the median duration from first care seeking until treatment initiation between patients accessing PPIA-E private facility at first point of care and those who did not. Other points of analysis included assessing the role of PPIA-E facilities in diagnosis and initiation of treatment, as well as the retention of the patients from the beginning of the pathway.

Median pathway differences were compared using the Mann Whitney U-test with significance established at p-values ≤ 0.05. Significant difference in the proportion of patients was estimated using Chi-square.

Since patients in the end-line study were followed until their current TB treatment, descriptive analysis for patients presenting an extended pathway is included to understand the reasons for shopping post their first diagnosis and treatment initiation.

## Results

### Patient selection

The household survey identified 285 and 230 PTB patients in Patna and Mumbai, respectively, of which only 64 (22%) in Patna and 86 (37%) in Mumbai were included in the study. Enlisting rate was low in both cities; in Patna primarily due to patients not meeting the inclusion criteria (62%) (Refer to study design) and in Mumbai, due to patients being diagnosed with drug-resistant (DR) TB (20%). Additionally, the reasons for exclusion from the study are reflected in Table 1. Individual results of baseline and end-line surveys are available as *Underlying data* ^16, 17^.

**Table 1.** Reasons for patient exclusion in Patna and Mumbai.

Reasons for patient exclusion	Mumbai, n (%) (N = 230)	Patna, n (%) (N = 285)
DR TB Cases	46 (20)	3 (1.1)
Refusals	28 (12.2)	18 (6.3)
Inconsistent information	0 (0)	2 (0.7)
Irregular treatment	14 (6.1)	5 (1.7)
EPTB Cases	14 (6.1)	41 (14.4)
No TB documents	10 (4.3)	40 (14)
Diagnosed / treated 6 months before study	4 (1.8)	90 (31.6)
Diagnosed outside the city	8 (3.4)	0 (0)
Migration	13 (5.7)	18 (6.3)
Died	7 (3)	4 (1.4)
DS-TB self-reporting patients	86 (37.4)	64 (22.5)

### Demographics

Table 2 reflects the comparison of patient profiles in both cities at baseline and end-line. Though certain trends were observed, the differences were not found to be significant. Low socio-economic status was retained in the end-line studies for both cities and higher numbers of patients were unemployed in both the cities at end-line studies.

The proportion of minor patients aged 17 years and younger was higher in Patna when compared with the baseline ( Table 2).

**Table 2.** Patient characteristics.

Patient characteristics	Patna baseline, n (%) (N=64)	Patna end-line, n (%) (N=64)	Mumbai baseline, n (%) (N=76)	Mumbai end-line, n (%) (N=86)
**Age (years)**				
≤17	19 (30)	27 (42)	8 (11)	10 (12)
>18	45 (70)	37 (58)	68 (89)	76 (88)
**Gender**				
Male	33 (52)	27 (42)	46 (61)	42 (49)
Female	31 (48)	37 (58)	30 (39)	44 (51)
**Occupation**				
Student	13 (20)	21 (33)	9 (12)	14 (16)
Housewife/unemployed	23 (36)	18 (28)	40 (53)	29 (34)
Self employed	10 (16)	8 (12)	10 (13)	6 (7)
Daily wage/casual	6 (9)	7 (11)	6 (8)	15 (17)
Salaried	9 (14)	3 (5)	11 (14)	19 (22)
Retired	0 (0)	1 (2)	0 (0)	2 (2)
Not available-Undefined	3 (5)	6(09)	0 (0)	1 (1)
**Education**				
Illiterate	22 (34)	19 (30)	14 (18)	14 (16)
Literate but no formal education	7 (11)	4 (6)	0 (0)	1 (1)
Primary (<4th standard)	7 (11)	17 (26)	8 (11)	6 (7)
Secondary (<9th standard)	15 (23)	7 (11)	32 (42)	30 (35)
Senior secondary (SSC/HSC)	8 (13)	12 (19)	16 (21)	27 (31)
Graduate and above	3 (5)	2 (3)	4 (5)	7 (8)
Not applicable (children ≤5 yrs)	2 (3)	3 (5)	2 (3)	1 (1)
**Use of addictive substances**				
No	43 (67)	42 (66)	52 (68)	60 (70)
Yes	21 (33)	22 (34)	24 (32)	22 (25)
Don’t Know	0 (0)	0 (0)	0 (0)	4 (5)
**Chronic conditions**				
No	54 (84)	52 (81)	63 (83)	65 (76)
Yes	9 (14)	12 (19)	13 (17)	21 (24)
Not available	1 (2)	0 (0)	0 (0)	0 (0)

The proportion of income generators among the patients in Mumbai had increased at end-line (47%, n=40/86) as compared to 33% at baseline ^10^.

### Pathways to TB care

***First point of care***

Had patients heard about the PPIA initiative?

**Patna:** In total, 52% of the patients (33/64) in Patna were aware of the PPIA initiative; 21 patients from providers they accessed along their pathways and 12 patients through media and posters.

**Mumbai:** In Mumbai, 42% of the patients (n=36/86) were aware of the PPIA intervention in the community, with 32 reportedly being provided information by the providers accessed along their pathways. Only 4 patients had sourced their awareness through media and posters.

What were the first care seeking facilities that patients accessed?

Table 3a, b shows the facilities first accessed by patients after the onset of symptoms, in Patna and Mumbai respectively.

**Table 3.** Type of facilities accessed by patients in the TB care pathway in Patna (A) and Mumbai (B).

(A) Patna				
Patna	First care seeking, n (%) (N=64)	First diagnosis, n (%) (N=64)	Final diagnosis, n (%) (N=4)	Treatment initiation, n (%) (N=64)
**Type of facility**				
PPIA NE public	3 (5)	6 (9.5)	-	6 (9.5)
PPIA NE private	16 (25)	18 (28)	-	17 (26)
PPIA NE chemist	30 (47)	-	-	-
PPIA public	1 (2)	6 (9.5)	1 (25)	6 (9.5)
PPIA private	10 (15)	34 (53)	3 (75)	35 (55)
PPIA chemist	4 (6)	-		NA
(B) Mumbai				
Type of facility	First care seeking, n (%) (N=86)	First diagnosis, n (%) (N=86)	Final diagnosis, n (%) (N=35)	Treatment initiation, n (%) (N=86)
Public	18 (21)	25 (29)	10 (29)	28 (32)
PPIA NE private	33 (38)	16 (19)	3 (8)	3 (4)
PPIA NE chemist	4 (5)	-	-	-
PPIA spoke	28 (34)	23 (27)	1 (3)	3 (3)
PPIA hub	3 (2)	22 (25)	21 (60)	52 (61)

**Patna:** The proportion of patients seeking care from public facilities (6%, n=4/64) was very similar to that reported in the baseline study ^9^. However, the proportion of patients consulting a chemist (53%, n=34/64) was significantly more than the 25% reported in the baseline study (p=-0.001, chi square =10.64). Less than one-fourth (23%, n=15/64) of the patients had consulted a PPIA-E facility, and two-thirds of these (67%, n=10/15) a private facility. Frequently cited reasons for accessing a PPIA-E facility were as follows: good/effective medicines, someone known had been previously treated or were on treatment, and subsidized fees. Reasons for accessing a PPIA-NE private facility were as follows: close to home, good reputation of the provider, whereas for accessing a PPIA-NE public facility the reasons cited were free medicines or worsening of symptoms.

**Mumbai:** Close to three-fourths of the patients (74%, n=64/86) had sought help from a private facility; almost half of these (48%, n=31/64) had consulted a PPIA-E private facility. The proportion of patients accessing a public sector facility as their first point of care were 21% (n=18/86) in the end-line study.

How soon after developing symptoms did patients seek care?

**Patna:** The median time taken for all patients to first access care after onset of symptoms was 9.5 days ^1^ in Patna which was similar to the 9 days ^1^ reported for the baseline study ^9^. The median time taken for patients accessing a PPIA-E private facility at first point was 4 days as opposed to 10 ^# 1^ days for those accessing PPIA-NE private facility and 8 days ^1^ for those accessing a private facility at first point during baseline ( Table 4a).

**Table 4.** Pathway durations baseline & end-line for public, private and PPIA-engaged private facilities in Patna (A) and Mumbai (B). All figures are depicted in days as median (mean, standard deviation).

(A) Patna					
Patna Pathways Median (Mean, SD)	Baseline Private Facility at first point (N=60)	End-line PPIA-E private facility at first point (N=14)	End-line PPIA-NE Private facility at first point (N=46)	Baseline Public Facility at first point (N=4)	End-line Public facility at first point (N=4)
First care seeking duration (days)	8 (15,19.9)	4 (24.9, 40.7)	10 (16,22.8)	18.5 (17,5.2)	7.5 (9,9.6)
Diagnostic duration (days)	*9.5 (24.6,34.5)	9.5 (23.1,32.3)	*20.5 (40.2,52.5)	2.5 (3.5,3.2)	18 (30.3, 33)
Treatment duration (days)	0 (1.8,5.9)	0 (0.7, 1.3)	0 (4,20.7)	1 (2.5,3.8)	0.5 (1.5,2.4)
Total Pathway duration (days)	27.5 (41.4,40.5)	15 (48.6, 63)	40.5 (60.2,60)	21.5 (23,5.9)	38 (40.75, 30.3)
(B) Mumbai					
Pathways Median (Mean, SD)	Baseline Private facility at first point (N=53)	End-line PPIA-E Private facility at first point (N=31)	End-line PPIA-NE Private facility at first point (N=37)	Baseline Public facility at first point (N=23)	End-line Public facility at first point (N=18)
First care seeking duration (days)	*14 (21.6,28.5)	*4 (9.6, 13.6)	*4 (11,15.4)	15 (30.4,51.1)	7.5 (11, 11.8)
Diagnostic duration (days)	21 (21,50)	16 (30, 37.1)	27 (43.4, 64.6)	10 (23.4,33.4)	10 (26.9, 29.8)
Treatment duration (days)	2 (5.2,9.3)	3 (6.4, 9.8)	2 (4, 4.7)	*0 (3.4,11)	*5.5 (14.9, 32.8)
Total Pathway duration (days)	*46 (68,67.7)	*32 (45.9, 42.5)	43 (58, 63)	41 (57.2,64.3)	39 (52.8, 44.3)

*Denotes significance at p-value 0.05.

Only 4/64 patients approached a public facility at first point of care. The median time taken for the patients who approached the public facility was 7.5 days ^1^ at end-line compared to 18.5 days at baseline.

Frequently cited reasons for accessing a PPIA-E facility at end-line were as follows: good/effective medicines, someone known had been previously treated or was on treatment, and subsidized fees. Reasons for accessing a PPIA-NE private facility were as follows: close to home, good reputation of the provider, whereas for accessing a PPIA-NE public facility the reasons cited were free medicine or worsening of symptoms.

**Mumbai:** In Mumbai the time taken to access care had decreased significantly from 15 days at baseline ^10^ to 4 days ^1^ at end-line (p-value = 0.00).

No significant difference was seen in the first care seeking duration for patients accessing PPIA-E private facilities vs. patients accessing PPIA-NE private facilities at Mumbai; however, both the groups had a significantly shorter first care seeking pathway as compared to baseline ( Table 4b).

The commonly cited reasons for a majority of the patients accessing a PPIA-E or a PPIA-NE private facility at first point were similar: close to home or the availability of the family doctor who practiced there. The reasons cited for accessing the public sector included: availability of effective or subsidized medicines, recommendation of a family member, and positive feedback from another patient.

***TB diagnostic pathway***

**Patna:** Patients who had accessed a PPIA-E private facility took 9.5 days ^1^ to get diagnosed after first accessing care, which was similar to that for patients accessing a private facility as first point of care during the baseline study ^9^. However, those patients who accessed a PPIA-NE private facility as the first point of care took significantly longer to get diagnosed, i.e. 20.5 days (p < 0.05). A significantly higher proportion of patients who approached a PPIA-E facility at the first point of care were diagnosed without any delay as compare to those who accessed a PPIA-NE facility (64% vs 39%; Table 5a).

**Table 5.** Proportion of patients showing delay and no delay in Patna (A) and Mumbai (B) based on the type of facility at first point of care.

(A) Patna					
Diagnostic/treatment delay	Baseline private (n=60)	Baseline public (n=4)	End-line private (n=46)	End-line public (n=4)	End-line PPIA private (n=14)
**No diagnostic delay**	36 (60%)	4 (100%)	18 (39.1%)	2 (50%)	9 (64.3%)
**With diagnostic delay**	24 (40%)	0 (0%)	28 (60.9%)	3 (75%)	5 (35.7%)
**No treatment delay**	56 (93.4%)	3 (75%)	43 (93.5%)	4 (100%)	14 (100%)
**With treatment delay**	4 (6.6%)	1 (25%)	3 (6.5%)	0 (0%)	0 (0%)
(B) Mumbai					
Diagnostic/treatment delay	Baseline private (n=53)	Baseline public (n=23)	End-line private (n=37)	End-line public (n=18)	End-line PPIA private (n=31)
**No diagnostic delay**	22 (41.5%)	13 (56.5%)	11 (29.7%)	10 (55.6%)	15 (48.4%)
**With diagnostic delay**	31 (58.5%)	10 (43.5%)	26 (70.1%)	8 (44.4%)	16 (51.6%)
**No treatment delay**	41 (77.4%)	22 (95.7%)	28 (75.7%)	10 (55.6%)	23 (74.2%)
**With treatment delay**	12 (22.6%)	1 (4.3%)	9 (24.3%)	8 (44.4%)	8 (25.8%)

In total, 90% of patients who approached a PPIA-E private facility (non-chemist) at first point of care were diagnosed by the same facility as compared to only 19% of the patients who approached a PPIA-NE private facility (chi-square =12.56, p-value = 0.0004). In the entire pathway 63% (n=40/64) of the patients were first diagnosed by a PPIA-E facility ( Table 3a). A total of 77% percent (n=49/64) of patients had approached up to two facilities before approaching the facility which diagnosed them with TB.

**Mumbai:** Although not significant, the median diagnostic duration for patients accessing a PPIA-E facility as the first point of care was 16 days which was lesser than the 27 days for those who accessed a PPIA-NE private facility and the 19 days reported in the baseline study ^10^. Patients approaching a public facility at first point, however, showed the least diagnostic duration of 10 days ^1^ (p > 0.05) which was the same as that at baseline ( Table 4b).

Two of the three patients that had accessed a PPIA-Ehub and two of the 28 patients (7%) that had accessed a PPIA-E spoke as the first point of contact were diagnosed by them. Only 9 out of 33 (27%) patients who approached a PPIA-NE private facility were diagnosed at the same facility. More than half of the total patients (52%, n=45/64) were first diagnosed by a PPIA-E facility: 23 by a PPIA-E spoke and 22 by a PPIA-E hub ( Table 3b). Among those who accessed public facilities as first point of care, 44% (n=8/18) were retained for diagnosis and three (17%) were referred for diagnosis.

A higher proportion of patients (48%, n =15/31) obtained diagnosis within the stipulated time when they first approached a PPIA-E private facility as compared to a PPIA-NE private facility (30%, n=11/37); this was also higher compared to the 41% in the baseline study who approached a private facility ^9^. The highest proportion of patients (56%) who obtained diagnosis within the stipulated time were those who had approached a public facility as their first point of care, at both study time points ( Table 5b).

A total of 49% (n=42/86) of patients had approached up to two facilities before approaching the facility which diagnosed them with TB.

***TB treatment pathway***

What happens to patients after they are first diagnosed with TB?

**Patna:** After being diagnosed with TB for the first time, four out of the 64 patients (6%) from Patna were retested and re-diagnosed before being initiated on TB treatment ( Table 3a).

**Figure 1.**
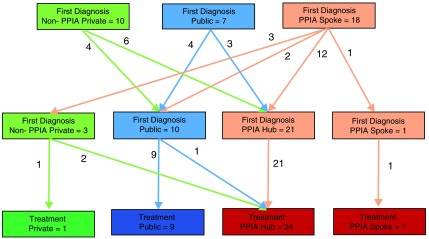
Facility switching for multiple TB diagnosis before treatment initiation in Mumbai.

**Mumbai:** Seeking and obtaining a second diagnosis was far more common in Mumbai. Forty-one percent (n=35/86) of the patients were retested and re-diagnosed before being initiated on TB treatment ( Table 3b). Figure 1 shows the facilities that re-diagnosed the patients before treatment initiation. Undergoing re-diagnosis was more commonly seen among patients who were initially diagnosed by a PPIA spoke (78%, n=18/23) or by a PPIA-NE facility (62%, n=10/16) and was less common among patients who had accessed the public facility (28%, n=7/25) for diagnosis. It was noteworthy that none of 22 patients diagnosed by a PPIA-E hub facility were re-diagnosed. Of the 35 (63%) final diagnoses, 22 were made by PPIA-E facilities. Some of the common reason for leaving the first diagnosing provider was referrals by the previous provider (71%) and patient seeking of a second opinion (11%).

After how long are patients being initiated on TB treatment after their first diagnosis?

**Patna:** In Patna, 95% (n=61/64) of the patients were initiated on TB treatment without any delay and 74% (n=45/61) of them on the same day. A total of 61% (n=37/61) of the patients who had no treatment initiation delay, were diagnosed by PPIA-E facilities, 29% (n=18/61) by a PPIA-NE private and 10% (n=6/61) by a PPIA-NE public facility. Only three patients in Patna had a delayed TB treatment initiation, all of who had been diagnosed by a PPIA-E facility (2 private and 1 public).

In total, 92% (n=59/64) of patients were initiated on treatment by their diagnosing facility. More patients approaching a PPIA-E private facility at first point of care were initiated on treatment by the same facility (80%, n=8/10) compared to those who approached a PPIA-NE private facility (19%, n=3/16). The rapidity of initiation of treatment was compromised for the four patients who had multiple diagnosis with a median of 10 days (Range: 3–140)

Of the 64 patients in Patna, 92% of patients were initiated on treatment by their diagnosing facility and only five patients moved to a different facility to initiate TB treatment.

Overall, 80% (n=51/64) of patients in Patna had accessed up to two facilities before initiating their TB treatment.

**Mumbai:** In total, 71% (n=61/86) of the patients from Mumbai were initiated on treatment without any delay, one-third of these (n=20/61) on the same day of diagnosis. The proportion of patients showing no treatment initiation delay was higher among those who were diagnosed by PPIA-E facilities (56%, n=34/61),compared to those diagnosed by public facilities (30%, n=17/61) and PPIA-NE facilities (16%, n=10/61). Paradoxically, among the 25 patients who had a treatment delay, the larger proportion had been diagnosed by PPIA-E facilities (44%, n=11/25), followed by PPIA-NE facilities (24%, n=6/25) and public facilities (32%, n=8/25).

Four of the 31 (13%) patients who had accessed the PPIA-E spoke (n=2)/hub facility (n=2) ( Table 3b) as the first point of care, were diagnosed and treated by the same facility.

No patient who had accessed a PPIA-NE private facility at first point was initiated on treatment by them. Of the 18 patients that had first approached a public facility, 6 (33%) were retained with the system. Close to two-thirds of the patients (55/86) had subsequent to their diagnosis, moved to another facility for initiating their treatment, of which 25% (14/55) had accessed more than two facilities after being diagnosed.

Two-thirds of the patients (62%, n=53/86) had approached up to three facilities before getting initiated on TB treatment after their diagnosis.

***Total TB pathway***

**Figure 2.**
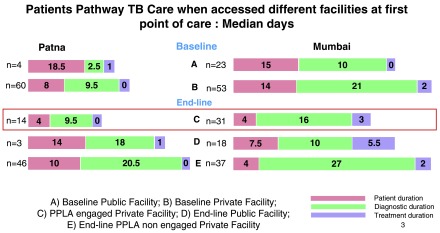
Patient total Pathways to TB Care in Mumbai & Patna.

**Patna:** The median total pathway duration (from onset of symptoms to initiation of first TB treatment) of patients in Patna was 15 days ^1^ for those accessing a PPIA-E private facility at first point of care, 38days for those accessing a public facility and 40.5 days for those accessing PPIA-NE private facility ( Figure 2). The pathway duration showed a reduction from the 27.5 days ^1^ for those accessing a private facility, but an increase from the 21.5 days ^1^ for those accessing a public facility in the baseline study ^9^. The total number of facilities accessed by the patients during their pathway ranged from 1–4 (median: 2).

**Mumbai:** In Mumbai, the total pathway duration was 43 days for patients accessing a PPIA-NE private facility at first point of care. There was a significant decrease in the median total pathway for patients accessing a PPIA-E facility (32 days) ^1^ at first point of care as compared to the patients accessing a private facility at baseline (46 days) (p<= 0.05). The duration for patients accessing a public facility was similar in the end-line and baseline studies (39 and 41 days, respectively) ( Figure 2). The total number of facilities accessed by the patients during their total care pathway at end-line ranged from 1–8 (median: 3).

### Extended pathway to TB care in the end-line study

***Does the patient pathway to TB care end once treatment is initiated?***

After being initiated on TB treatment for the first time, 10 out of the 64 patients in Patna (16%) and 9 out of the 86 patients in Mumbai (10%) left the treating facilities and showed an extended pathway in the end-line study. This was further looked into to understand what made these patients leave their treating facilities.

**Figure 3.**
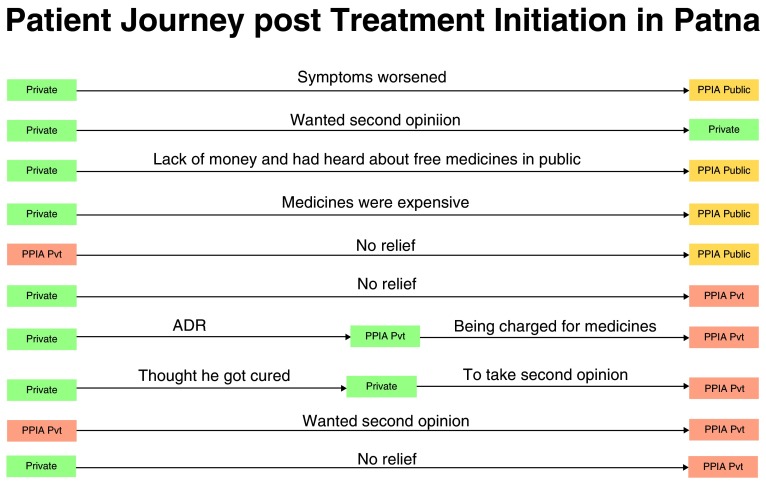
The extended pathway for patients in Patna.

**Patna:** From the 4% (n=2/51) of patients who were initiated on treatment by PPIA-E facilities and 62% (n=8/13) who were initiated on treatment by a PPIA-NE facility had an extended pathway. The median pathway duration for patients from first care seeking until first treatment initiation was 38 days (mean: 41 days, range: 5-79 days) which was extended by an additional 49 days (mean: 72 days, range: 5-187 days) due to the multiple treatment episodes. Figure 3 presents the reasons for the extended pathway for patients in Patna.

**Figure 4.**
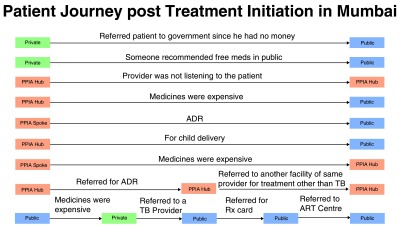
The extended pathway for patients in Mumbai.

**Mumbai:** A total of 9 out of the 86 patients from Mumbai had an extended pathway. This group with extended pathways constituted 11% (n=6/55) of those initiated on treatment by a PPIA-E facility, 66% (n=2/3) of those initiated on treatment by a PPIA-NE facility and 4% (n=1/28) of those initiated on treatment by a public facility. The total median pathway duration for these patients from first care seeking until first treatment initiation was 62 days (mean: 50 days, range: 2-206 days). However, the median duration between first TB treatment initiation and current/final TB treatment initiation was an additional 28 days (mean: 34 days, range: 3-70 days). Figure 4 presents the reasons for the extended pathway for patients in Mumbai.

## Discussion

Public–private partnerships (PPP) have been implemented globally to improve access, care and support for TB patients accessing private health care. Many interventions have been evaluated for increase in case detection rates ^18, 19^ and few for cost effectiveness ^8^. Our end-line study undertaken in the two cities of Mumbai and Patna to evaluate the effect of a PPIA intervention on durations and delays in TB care is, to our knowledge, the first of its kind.

The initiative at the two sites had their own distinct flavours. Firstly, the model was played out differently in the two cities. Mumbai practised a spoke and hub referral model, whereas in Patna a one-stage approach (sans referrals) prevailed. Only private practitioners participated in the initiative in Mumbai whereas in Patna, public practitioners also engaged with the PPIA as private system providers. Molecular diagnostic CBNAAT technology was readily available in Mumbai with 28 instruments operational, whereas Patna during the evaluation was witnessing a relatively slow uptake of the CBNAAT (three pieces of equipment installed). Lastly, in Patna there was relatively more effort to make its citizens aware of PPIA engagement with private providers. (WHP and PATH Personal Communication).

Delays in diagnosis and initiation of treatment have been of concern for patients seeking private care ^20, 21^. The PPIA intervention in Patna and Mumbai was precisely designed to achieve early diagnosis and ensure prompt initiation of appropriate treatment. We discuss to what extent the PPIA has been effective in reducing diagnostic and treatment delays and encouraging patient retention in uncomplicated PTB in both cities. The analytical framework includes comparisons of patient pathway durations between PPIA-E and PPIA-NE providers in both private and public at end-line as well as durations recorded at baseline prior to the PPIA initiation. The small sample size contributes to the quantitative data, while able to portray trends for durations and retentions, was unable to provide statistical significance to many comparisons.

In both the cities, as documented by most studies from India ^22– 25^, the private sector (including chemists in Patna) was predominantly accessed by patients at first point of care. The PPIA intervention did not specifically involve community engagement measures. Therefore, despite moderate attempts at community engagement in Patna, the study observed that a low proportion of patients (23%) accessed PPIA-E facilities at first point of care. Even those that accessed PPIA-E facilities had approached these randomly probably due to proximity to their residence, as seen from the reasons that patients provided for choosing their first facility. The other reason for the smaller proportions of patients consulting a PPIA-E facility could also be due to the less than optimal coverage of the private practitioners by the PPIA, not necessarily in terms of geographic area, but also the proportion of private practitioners engaged and the asynchronous locations of patients and practitioners. (Patna: 71%, Mumbai: 41%) (Personal Communication, WHP and PATH). Expansion of the base of engaged practitioners and designing approaches to raise community awareness to PPIA and its benefits, and also incorporating content to improve disease and treatment literacy ^26^ becomes an important message for similar initiatives in the future. This is brought out strikingly in Mumbai which showed a significant decrease in first care-seeking duration attributable to the intense awareness drives undertaken by the public system, especially after 2016. An informed patient can be a more adherent patient achieving better outcomes. Paradoxically, despite obvious implications for patient outcome, information education and communication/advocacy communication and social mobilization activities are categorised as those that have no indicators and are therefore not measurable. This viewpoint has led to cumulative ignorance about TB globally. No wonder then that at the recent UN High Level Meetings in September 2018, minimal support was allocated for IEC activities ^27^.

Our study clearly shows that patients in both cities, who first approached PPIA-E facilities, took considerably shorter time to get diagnosed. Retention of patients after first contact was also satisfactory at PPIA-E facilities at first point of care, with figures of 90% and 67% at Patna and Mumbai, respectively. The retention in Mumbai was only seemingly affected by the spoke-hub referral where the former often referred their patients to a hub for diagnosis.

The public facilities in Mumbai however showed the least diagnostic duration when approached at first point of care though retention of patients after first contact was compromised. A higher proportion of patients approaching a PPIA-E facility showed no delay in diagnosis (64% and 48% in Patna & Mumbai respectively) when compared to a PPIA-NE facility. Timely diagnosis in Mumbai at public facilities consulted at first point of care remained constant at baseline ^10^ and end-line showing a sustenance in its efficacy.

Patient movement after obtaining first diagnosis was noted in both cities, although it was far more common in Mumbai with its vast numbers and types of private practitioners. While in Patna, patients gravitated from PPIA-NE to PPIA-E facilities, in Mumbai, PPIA-E spoke patients witnessed the most movement due to the mandatory process of a second diagnostic procedure largely from a PPIA hub. A significant proportion of patients who were moved to the public sector (25%) and the PPIA-NE facilities (63%) for first diagnosis were subjected to re-diagnosis. Thus, the patients from the PPIA-NE facilities showed the largest movement after the first diagnosis, with PPIA-E hub patients showing a strong tendency to remain with their providers.

The triggers for patient movement were manifold, namely dissatisfaction with provider, denial of diagnosis, recommendations from friends or relatives or even referrals by providers themselves. These require to be better understood in different contexts through an explanatory model framework ^28^.

After initiation of treatment by their diagnostic facility, movement of patients was minimal. The largest proportion of patients that showed no delay in initiation of treatment in both cities were from the PPIA-E segment. Paradoxically in Mumbai the largest proportion of patients showing delay had also been diagnosed by a PPIA-E facility. Patients with multiple diagnostic episodes also showed the longest treatment initiation pathways. Retention of the patient at first point of care to treatment initiation was weak even in those who had accessed PPIA-E facilities.

The limitations of small sample sizes and possible recall bias notwithstanding, our findings show that accessing a PPIA-E facility at first point of care undoubtedly shortened the total care pathway in both cities. In contrast, patients who accessed non-engaged private providers at first point of care had the longest pathways. These are observations consistent with previous findings that the type of provider first consulted was the most important risk factors for delays ^12^ and that patients who accessed private non-engaged facilities had longer downstream delays ^22^.

The issue of extended pathways to care beyond initiation of treatment in 10–15% of patients in both cities was an additional component of the end-line study. The majority of those who demonstrated extended pathways in Patna moved from the PPIA NE sector to the PPIA-E/public facilities. Mumbai provided a contrast since the extended pathway was noted in largely PPIA-E patients (hub and spoke) with most patients finally accessing public facilities. Dissatisfaction with the physician expenses and inability to address adverse drug reactions were reported by patients as key reasons.

The number of providers accessed for DS-TB in Patna and Mumbai ranged from 1–4 and 1–8, respectively. Minimizing patient movement would require a uniform standard of care ^29^ that could, over time, generate implicit trust among patients for the provider accessed. This coupled with enhanced disease literacy would shorten patient delay significantly. Costs of drugs and consultation are often a significant cause of movement from the private to the public sectors ^22^. Therefore extending the free availability of diagnostics and drugs to private patients as in the Joint Effort for Elimination of TB (JEET) programme ^30^ is expected to immensely benefit patients, provided the public system is able to retain the inter-sectoral patients by providing good quality services at a reasonable consultation which could be regulated.

The issue of provider quality of care in terms of diagnosed tests applied and treatment prescribed was, however, not examined in this study. Amongst the PPIA-E providers also, other quality indicators such as delays, retentions were not absolute. Although deviant delays/movement could also be an outcome of patient behaviour, any future public private initiative should include frequent sensitization of providers so that they are capable of managing treatment and adverse reactions. Though we have no data to support this, communication skills of provider and empathetic behaviour (such as time spent with patient) would be useful add-ons as would the provision of free/subsidized diagnostics and medication.

Additionally, the design of any PPI should be to lessen patient movement. Movement reduces energy and motivation in an already vulnerable patient. Providers and ancillary diagnostic/path services should, as far as possible, be proximal to patient as well as provider location to avoid delays and sustain treatment motivation ^31^.

 The present study provides a strong indication of the utility of public-private system interactions to tackle drug sensitive pulmonary TB and provides guidance to move further on the pathways of patient centred care ^32^. Certain key lessons emerge from this study for strategic involvement of the private sector. These are to monitor progress, engage maximum providers, and build their capacities to offer standard quality of care. Finally, as evinced in both Mumbai and Patna, flexibility of PPM structures to suit local context and dynamics is a successful strategy.

## Data availability

### Underlying data

Figshare: Mumbai Patna Baseline data. https://doi.org/10.6084/m9.figshare.11949117.v1 ^16^.

This project contains the following underlying data:

- Mumbai_BL_DS_TB cases (XLSX). (Baseline household survey data for participants in Mumbai.)
- Patna_BL_DS_TB cases (XLSX). (Baseline household survey data for participants in Patna.)

Figshare: Mumbai Patna Endline data. https://doi.org/10.6084/m9.figshare.11949147.v1 ^17^.

This project contains the following underlying data:

- Mumbai_EL_DS_TB cases (XLSX). (Endline household survey data for participants in Mumbai.)
- Patna_EL_DS_TB cases (XLSX). (Endline household survey data for participants in Patna.)

The transcripts and audio recordings of the interviews cannot be shared as patient identifiers cannot be removed. However, this information can be requested from the Institutional Ethics Committee. The decision will be taken on case to case basis. Queries and requests for data access should be directed to The Foundation for Medical Research Institutional Review and Ethics Committee at fmr-irec@fmrindia.org. Granting of access of data will be at the discretion of the FMR-IREC.

### Extended data

Figshare: Supplementary Data. https://doi.org/10.6084/m9.figshare.12045867.v1 ^13^.

This project contains the following extended data:

- BMGF Codebook Mumbai (XLSX). (Codebook for Mumbai surveys.)
- BMGF Codebook_patna (XLSX). (Codebook for Patna surveys.)
- BMGF_Mumbai Datasheet Questnr (DOCX). (Survey questionnaire for Mumbai.)
- BMGF_Patna Datasheet Questnr (DOCX). (Survey questionnaire for Patna.)
- Final_interview_Guide_Mum (PDF). (Interview guide for Mumbai.)
- Final_interview_Guide_Patna (PDF). (Interview guide for Patna.)

Unrestricted data are available under the terms of the Creative Commons Zero "No rights reserved" data waiver (CC0 1.0 Public domain dedication).

## Notes

^1^Durations which fall within the standard norms of care

## References

[1] Programme Rnt: National Stratergic Plan for Tuberculosis Elimination 2017-2025.2017 Reference Source

[2] Organisation WH: Global tuberculosis report. Geneva: World Health Organization;2017 Reference Source

[3] Chauhan L: Drug resistant TB--RNTCP response. *Indian J Tuberc.* 2008;55(1):5–8. 18361304

[4] Dholakia Y Mistry N: Active tuberculosis case finding in a migrant slum community, Mumbai, India. *Int J Tuberc Lung Dis.* 2016;20(11):1562. 10.5588/ijtld.16.0722 27776601

[5] Aye S Majumdar SS Oo MM: Evaluation of a tuberculosis active case finding project in peri-urban areas, Myanmar: 2014-2016. *Int J Infect Dis.* 2018;70:93–100. 10.1016/j.ijid.2018.02.012 29476901

[6] Kulshrestha N Nair SA Rade K: Public-private mix for TB care in India: Concept, evolution, progress. *Indian J Tuberc.* 2015;62(4):235–8. 10.1016/j.ijtb.2015.11.003 26970466

[7] Organisation WH: Public-Private Mix (PPM) for TB Care and Control.2019 Reference Source

[8] Floyd K Arora V Murthy K: Cost and cost-effectiveness of PPM-DOTS for tuberculosis control: evidence from India. *Bull World Health Organ.* 2006;84(6):437–45. 10.2471/blt.05.024109 16799727 PMC2627367

[9] Mistry N Lobo E Shah S: Pulmonary tuberculosis in Patna, India: Durations, delays, and health care seeking behaviour among patients identified through household surveys. *J Epidemiol Glob Health.* 2017;7(4):241–8. 10.1016/j.jegh.2017.08.001 29110864 PMC7384577

[10] Mistry N Rangan S Dholakia Y: Durations and delays in care seeking, diagnosis and treatment initiation in uncomplicated pulmonary tuberculosis patients in Mumbai, India. *PLoS One.* 2016;11(3):e0152287. 10.1371/journal.pone.0152287 27018589 PMC4809508

[11] Saqib MA Awan IN Rizvi SK: Delay in diagnosis of tuberculosis in Rawalpindi, Pakistan. *BMC Res Notes.* 2011;4(1):165. 10.1186/1756-0500-4-165 21615946 PMC3123219

[12] Sreeramareddy CT Qin ZZ Satyanarayana S: Delays in diagnosis and treatment of pulmonary tuberculosis in India: a systematic review. *Int J Tuberc Lung Dis.* 2014;18(3):255–66. 10.5588/ijtld.13.0585 24670558 PMC4070850

[13] Medical Research: Foundation for Medical Research (2020): Supplementary Data. figshare. Dataset. 10.6084/m9.figshare.12045867.v1

[14] Organisation SFTCWH: World Health Organization. Standards for TB care in India. World Health Organization, Geneva, Switzerland. 2014.2014 Reference Source

[15] Control CTDTaOgFT: Central Tuberculosis Division Technical and Operational guidelines For Tuberculosis Control.2005 Reference Source

[16] Medical Research: Foundation for Medical Research (2020): Mumbai Patna Baseline data. figshare. Dataset. 10.6084/m9.figshare.11949117.v1

[17] Medical Research: Foundation for Medical Research (2020): Mumbai Patna Endline data. figshare. Dataset. 10.6084/m9.figshare.11949147.v1

[18] Arora VK Sarin R Lönnroth K: Feasibility and effectiveness of a public-private mix project for improved TB control in Delhi, India. *Int J Tuberc Lung Dis.* 2003;7(12):1131–8. Reference Source 14677887

[19] Lestari BW Arisanti N Siregar AY: Feasibility study of strengthening the public–private partnership for tuberculosis case detection in Bandung City, Indonesia. *BMC Res Notes.* 2017;10(1):404 10.1186/s13104-017-2701-y 28807020 PMC5557311

[20] Pai M: Promoting affordable and quality tuberculosis testing in India. *J Lab Physicians.* 2013;5(1):1 10.4103/0974-2727.115895 24014959 PMC3758696

[21] Selvam Paramasivam BT Chandran P Thayyil J: Diagnostic delay and associated factors among patients with pulmonary tuberculosis in Kerala. *J Family Med Prim Care.* 2017;6(3):643. 10.4103/2249-4863.222052 29417023 PMC5787970

[22] Veesa KS John KR Moonan PK: Diagnostic pathways and direct medical costs incurred by new adult pulmonary tuberculosis patients prior to anti-tuberculosis treatment–Tamil Nadu, India. *PLoS One.* 2018;13(2):e0191591. 10.1371/journal.pone.0191591 29414980 PMC5802859

[23] Arinaminpathy N Batra D Khaparde S: The number of privately treated tuberculosis cases in India: an estimation from drug sales data. *Lancet Infect Dis.* 2016;16(11):1255–60. 10.1016/S1473-3099(16)30259-6 27568356 PMC5067370

[24] Sudha G Nirupa C Rajasakthivel M: Factors influencing the care-seeking behaviour of chest symptomatics: a community-based study involving rural and urban population in Tamil Nadu, South India. *Trop Med Int Health.* 2003;8(4):336–41. 10.1046/j.1365-3156.2003.01010.x 12667153

[25] Rajeswari R Chandrasekaran V Suhadev M: Factors associated with patient and health system delays in the diagnosis of tuberculosis in South India. *Int J Tuberc Lung Dis.* 2002;6(9):789–95. 12234134

[26] Mbuthia GW Olungah CO Ondicho TG: Health-seeking pathway and factors leading to delays in tuberculosis diagnosis in West Pokot County, Kenya: A grounded theory study. *PLoS One.* 2018;13(11):e0207995. 10.1371/journal.pone.0207995 30485379 PMC6261612

[27] Kamugasha RP: Innovation is key to #endTB: A disease riddled with myths [Press release]. Uganda: Citizen News Service; 2018 [updated August 28,2018 Reference Source

[28] Weiss MG: The promise of cultural epidemiology. *Taiwanese Journal of Psychiatry.* 2017;31(1):8–24. Reference Source

[29] Pai M Temesgen Z: Quality: The missing ingredient in TB care and control. *J Clin Tuberc Other Mycobact Dis.* 2019;14:12–3. 10.1016/j.jctube.2018.12.001 31720411 PMC6830165

[30] William J: Clinton Foundation CfHRaI, PATH, Foundation for Innnovative New Diagnostics. JOINT EFFORT FOR ELIMINATION OF TUBERCULOSIS (JEET). FIND, editor. Media. Online: William J. Clinton Foundation , Centre for Health Research and Innovation, PATH, Foundation for Innnovative New Diagnostics;2018 Reference Source

[31] Hanson C Osberg M Brown J: Finding the missing patients with tuberculosis: lessons learned from patient-pathway analyses in 5 countries. *J Infect Dis.* 2017;216(suppl_7):S686–S95. 10.1093/infdis/jix388 29117351 PMC5853970

[32] Organisation WH: First-ever targeted roadmap outlines steps to scale-up public-private health sector engagement to end TB The Hague: WHO;2018 Reference Source

