# Supplementary material for: Effect of public-private interface agency in Patna and Mumbai, India: Does it alter durations and delays in care seeking for drug-sensitive pulmonary tuberculosis?
Source: Gates Open Res. 2020 Apr 9;4:32. [Version 1] doi: 10.12688/gatesopenres.13113.1 (PMC7221294; doi:10.12688/gatesopenres.13113.1)
Supplement: Peer review history

# Peer review: Reviewer response for version 1

**DOI:** 10.21956/gatesopenres.14288.r28765

**Published:** 2020-05-13

**Authors:** Andrew McDowell

**Affiliations:** 1 Department of Anthropology, Tulane University, New Orleans, LA, USA

This article presents a vast array of carefully collected and curated data. To my mind it makes three important contributions to the conversation about innovative tuberculosis (TB) interventions in India and other TB-affected communities. First, it presents a unique mode for assessing programmatic interventions. Second, it highlights the combined importance of work to reduce delay and provide quality treatment. Third, it compares the private sector (intervention acceptors and non-acceptors) with public sector care. Though at times a difficult read the report of this study is crucial for public health actors, intervention scientists, and social sciences of heath researchers worldwide. 

 First, the paper presents a new paradigm for assessing and comparing the effect of a particular public health intervention. In this case the authors look to a public private partnership aimed at reducing rates of TB and improving the quality of its care in two Indian cities. By conducting a baseline and endline patient pathway survey the team is able to show an effect on the speed to diagnosis and other desirable outcomes like reduced provider switching, stronger referral processes, shorter periods before first care seeking and reductions in treatment delay. They are also able to identify troubling effects like unexplained re-testing. These findings in and of themselves provide an important set of metrics with which to understand the effects of an intervention. They are also comparable across space and time, so they allow for comparisons between interventions as well as no intervention. This kind of metric is a crucial tool because it is a measure of success and failure which can provide data more quickly than waiting for a population level impact on incidence and prevalence. Though population level mortality and morbidity reduction shown through incidence and prevalence trends will remain the goal, this paper presents a tool to understand and assess the patient and community-level effects that will bring about a shift in population indicators across time. 

 Second, the paper presents novel data on what happens to patients after treatment initiation. They do so by considering the shifts people make between care providers rather than a single-minded attention to treatment adherence. The show, what we ought know but often forget, that patient care extends past treatment initiation and that patient-provider relationships continue to matter for successful TB care at the individual and population level. Figures 3 and 4 show that, despite the free provision of TB drugs in the PPIA-engaged private sector, the cost of medicines continues to burden patients. This is a crucial corrective to PPIA engagement programs and suggests we pay better attention to patient costs regardless of sector. If patients are moving from the private to the public sector as these data indicate, it suggests that either physicians are supplementing their TB treatments with expensive pharmaceuticals or their consultations fees across the duration of care are burdensome. Nonetheless the data show that patients moved from non-PPIA-accepting private physicians to both PPIA accepting and public sector care. This is a heartening data point which shows that patients once inside the PPIA system did not leave it for other private sector physicians. These suggestive data points open yet another avenue by which to consider the effect of intervention. 

 Finally, the paper is part of a very small body of work that has the data to compare public and private sector indicators. Their data strongly suggests that the public sector continues to be an important source of reliable care. They show that without intervention the public sector (in Mumbai at least) was the best performing in terms of diagnostic delay. They also show that the engaged private sector outperforms the public when it comes to time from diagnosis to treatment initiation. The research’s innovative design has thus provided two action points: 1) build a PPIA system in which first point of care is a PPIA engaging physician and 2) work within the public sector to streamline diagnosis to treatment initiation. Overall their results show that the public sector, in Mumbai at least, continues to be an important source of TB care and our collective effort to engage the private sector should not be considered as an either/or initiative but ought in tandem with support to the public sector. 

 As an anthropologist working for over 10 years with TB patients in India, I found myself busy underlining this text and writing exclamation in its margins. Most exclamations were “wow” and “why?”. Though out of the scope of this paper, I was left wanting to learn more about the kinds of events that led to surprising findings like 25% of patients visiting 2 or more facilities after diagnosis, or why rates of retesting were so high in both cities, or what might explain why only 19% of non-accepting private clinics in Patna were able to retain their patients through diagnosis. These stunning findings are important insights and it is clear that the researchers probed their interviewees about the why’s and how’s of their treatment journey. However, the form they chose privileges quantitative trends over an analysis of patient actions and meanings. That said they open many new avenues for qualitative research, and I would encourage them to conduct an qualitative analysis of their data. 

 Finally, I wonder, as Prof. Daftary does, about the effects of their inclusion criteria on the broader evidentiary power of their results. Certainly, these criteria allow for the kinds of comparability I lauded above, but they raise questions about what we can take from this study as data about the state of all TB care and care seeking in each of these cities. By removing non-pulmonary forms of TB, patients without a diagnostic report or microbiological confirmation, and those suffering from MDR-TB from their sample, they remove a large diversity of people and events that make up the TB landscape in India. Luckily the authors do not make claims about the landscape in each city but make rather modest assertions about indicators of change within this smaller population due to an intervention. Readers might keep in mind that their study, though deep, tells us about one manifestation of TB among many. 

 Overall, this is a laudable study that maintains and exceeds expectations of methodological and analytical rigor. The authors have made claims clearly based in the evidence they present here and help readers think though innovative ways to understand public health action at intermediate scales. Their manuscript merits indexing.

Is the work clearly and accurately presented and does it cite the current literature?

Yes

If applicable, is the statistical analysis and its interpretation appropriate?

I cannot comment. A qualified statistician is required.

Are all the source data underlying the results available to ensure full reproducibility?

No source data required

Is the study design appropriate and is the work technically sound?

Yes

Are the conclusions drawn adequately supported by the results?

Yes

Are sufficient details of methods and analysis provided to allow replication by others?

Yes

Reviewer Expertise:

Anthropology, health seeking, South Asia, tuberculosis, global health, the private sector, diagnosis and care.

I confirm that I have read this submission and believe that I have an appropriate level of expertise to confirm that it is of an acceptable scientific standard.

# Peer review: Reviewer response for version 1

**DOI:** 10.21956/gatesopenres.14288.r28767

**Published:** 2020-05-07

**Authors:** Amrita Daftary

**Affiliations:** 1 School of Health Policy and Management, York University, Toronto, Canada

This paper articulates in remarkable detail the nuances of TB care seeking, diagnosis and treatment initiation in the context of a PPIA initiative in two urban centres of India. The research team has highlighted how complex the pathway to TB care can be and especially highlighted the continuous shuffling that patients undergo even when an apparently more seamless linkage to services has been attempted.

 There are lot of results and comparisons to sift through. Figure 2 is the most helpful summation as it addresses the primary research questions that the authors seek to address. It would be helpful in the discussion to very succinctly tease that finding apart, so readers can fully appreciate the benefit (and lack thereof - where no apparent effect or a negative effect was noted) of engaging private providers in each city, what appeared to work / fail in each city (and why, if possible). This would be a main take home message of the paper, in my opinion, and could guide future goals of PPIA programs (e.g., that reduction in diagnostic delay is indeed a feasible and attainable goal, with certain caveats / unknowns)

 An important outcome of the PPIA appears to be a reduction in patient delay (again, from Fig 2) vs impacts on other types of delays. One important implication that the authors could consider raising is the opportunity to educate and support presumptive patients about TB infection control at the first point of contact. The opportunity brought on by an early patient-provider interaction should not be missed, particularly given greater awareness about social distancing and isolation in current times. Whether or not potential patients abide by these recommendations is of course a separate matter but if timely TB diagnosis is aimed to prevent transmission and reduce incidence (alongside direct patient impacts), then the gains of reduced patient delay should be capitalized on.

 The eligibility criteria as displayed in text compared to patients excluded in Table 1 are not intuitively understood. Suggest adding a brief statement in text about who was excluded.

 Over/about 2/3 of the target sample was excluded from the study for a number of (justifiable) reasons. That being said, it would be of interest for readers to learn the authors' opinions about how those missed pathways could contribute to our understanding of patient pathways more broadly, less so around DRTB and EPTB but more so around migration, self reporting patients (20-35% of the target sample) who likely did attempt to access care from a provider if the questions were probed, and persons having no TB documents. As these factors all contribute to the complex reality in which many patients seek medical care. In future work, for example, would some of these patients be included?

 I have otherwise no substantial comments on the analysis. Thank you for the opportunity to review this paper.

Is the work clearly and accurately presented and does it cite the current literature?

Yes

If applicable, is the statistical analysis and its interpretation appropriate?

Yes

Are all the source data underlying the results available to ensure full reproducibility?

Yes

Is the study design appropriate and is the work technically sound?

Yes

Are the conclusions drawn adequately supported by the results?

Yes

Are sufficient details of methods and analysis provided to allow replication by others?

Yes

Reviewer Expertise:

tuberculosis, social and behavioural research, private sector, patient perspectives, qualitative methods

I confirm that I have read this submission and believe that I have an appropriate level of expertise to confirm that it is of an acceptable scientific standard.
